# Supplementary material for: Genomic evidence of adaptive evolution in the reptilian SOCS gene family
Source: PeerJ. 2021 Jun 24;9:e11677. doi: 10.7717/peerj.11677 (PMC8236234; doi:10.7717/peerj.11677)
Supplement: Supplemental Information 3 — The “Test” column shows the results of dS-dN, and p < 0.01 indicates the significant negative selection; the “GARD” column shows the results of recombination. [file peerj-09-11677-s003.docx]

| Gene | Test | p-value | GARD |
| --- | --- | --- | --- |
| *CISH* | 7.089 | 0.000 | None |
| *SCOS1* | 10.096 | 0.000 | None |
| *SCOS2* | 7.079 | 0.000 | None |
| *SCOS3* | 8.285 | 0.000 | None |
| *SCOS4* | 12.517 | 0.000 | None |
| *SCOS5* | 11.870 | 0.000 | None |
| *SCOS6* | 14.670 | 0.000 | None |
| *SCOS7* | 8.485 | 0.000 | None |
